# Indirect Effects of Conservation Policies on the Coupled Human-Natural Ecosystem of the Upper Gulf of California

**DOI:** 10.1371/journal.pone.0064085

**Published:** 2013-05-15

**Authors:** Hem Nalini Morzaria-Luna, Cameron H. Ainsworth, Isaac C. Kaplan, Phillip S. Levin, Elizabeth A. Fulton

**Affiliations:** 1 Marine Resources Assessment Group Americas Incorporated, Seattle, Washington, United States of America; 2 Conservation Biology Division, Northwest Fisheries Science Center, National Marine Fisheries Service, National Oceanic and Atmospheric Administration, Seattle, Washington, United States of America; 3 The Commonwealth Scientific and Industrial Research Organisation Wealth from Oceans Flagship, Division of Marine and Atmospheric Research, Hobart, Tasmania, Australia; University of Hamburg, Germany

## Abstract

High bycatch of non-target species and species of conservation concern often drives the implementation of fisheries policies. However, species- or fishery-specific policies may lead to indirect consequences, positive or negative, for other species or fisheries. We use an Atlantis ecosystem model of the Northern Gulf of California to evaluate the effects of fisheries policies directed at reducing bycatch of vaquita (*Phocoena sinus*) on other species of conservation concern, priority target species, and metrics of ecosystem function and structure. Vaquita, a Critically Endangered porpoise endemic to the Upper Gulf of California, are frequently entangled by finfish gillnets and shrimp driftnets. We tested five fishery management scenarios, projected over 30 years (2008 to 2038), directed at vaquita conservation. The scenarios consider progressively larger spatial restrictions for finfish gillnets and shrimp driftnets. The most restrictive scenario resulted in the highest biomass of species of conservation concern; the scenario without any conservation measures in place resulted in the lowest. Vaquita experienced the largest population increase of any functional group; their biomass increased 2.7 times relative to initial (2008) levels under the most restrictive spatial closure scenario. Bycatch of sea lions, sea turtles, and totoaba decreased > 80% in shrimp driftnets and at least 20% in finfish gillnet fleets under spatial management. We found indirect effects on species and ecosystem function and structure as a result of vaquita management actions. Biomass and catch of forage fish declined, which could affect lower-trophic level fisheries, while other species such as skates, rays, and sharks increased in both biomass and catch. When comparing across performance metrics, we found that scenarios that increased ecosystem function and structure resulted in lower economic performance indicators, underscoring the need for management actions that consider ecological and economic tradeoffs as part of the integrated management of the Upper Gulf of California.

## Introduction

Management of natural resources includes setting limits on exploitation or setting aside areas as reserves [1]. In marine systems, fisheries management aims to ameliorate the negative effects of fishing (i.e. population collapse, bycatch of non-target species, reduced habitat complexity, altered predator-prey relationships) through actions such as closed seasons and areas, limited entry, and gear restrictions [2]–[5]. Fisheries policies often regulate particular fleets or gears in an effort to protect species of conservation concern, such as marine mammals, sea turtles, and birds (i.e. [6]–[8]). However, species- or fishery- specific policies may lead to indirect consequences, both positive and negative, for other species, fisheries or whole ecosystems [9]. For example, closures could shift fishers into areas with higher bycatch of more vulnerable species or size classes [10] and length limits could actually expose target species and the ecosystem to increased negative effects [11].

**Figure 1:**
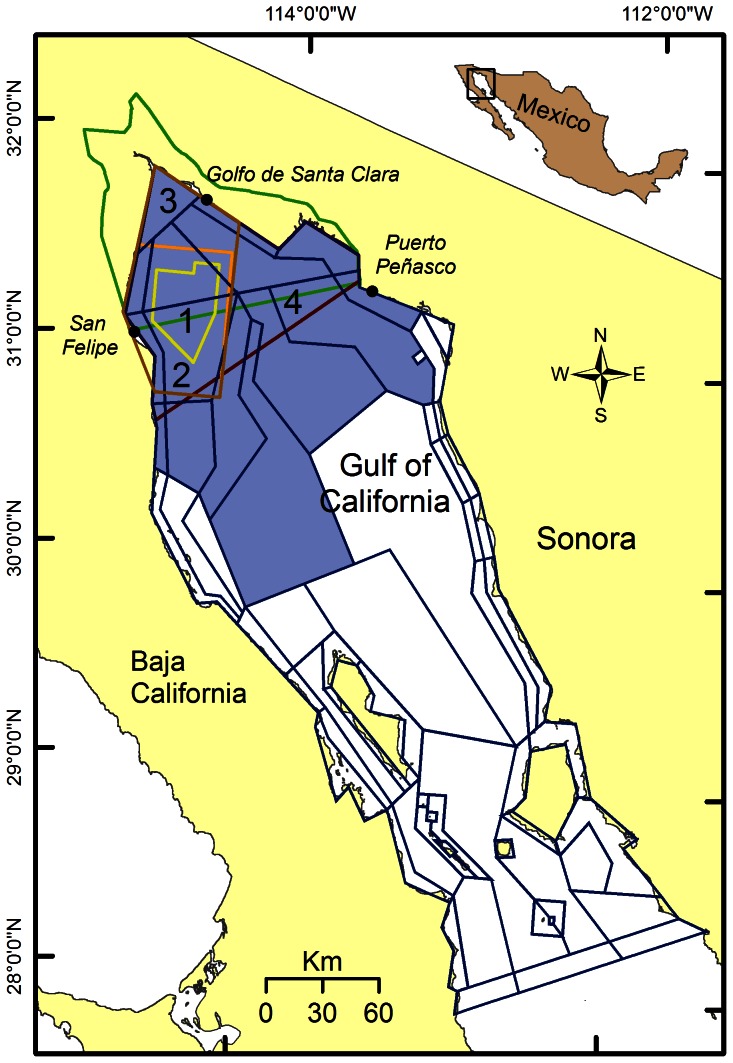
Northern Gulf of California Atlantis model extent, including Atlantis polygon geometry and polygons affected by management scenarios (blue shading). The main fishing communities in the Upper Gulf are indicated. Simulated spatial management restrictions in the Upper Gulf (yellow to red lines), including the Upper Gulf Biosphere Reserve (green line). Numbers correspond to scenarios: 1) Vaquita refuge (1264 km^2^), 2) Extended refuge (3579 km^2^), 3) Primary area (5339 km^2^) and 4) Distribution area (8432 km^2^).

Indirect consequences of bycatch reduction measures are evident in policies directed at vaquita (*Phocoena sinus*), a Critically Endangered [12] porpoise endemic to the Upper Gulf of California that is frequently entangled in finfish gillnets and shrimp driftnets. The vaquita population has declined rapidly from an estimated 567 individuals in 1997 (95% CI 177-1073; [13] to 245 in 2008 (95% CI 68-884; [14]. In 2009, the instantaneous annual bycatch mortality rate was high, 0.07 year^−1^ (7%), despite the implementation of bycatch reduction measures [14]. To protect vaquita, the Mexican government initially established the Upper Gulf of California and Colorado River Delta Biosphere Reserve [15] (See Methods for more details). However, a subsequent survey of vaquita distribution [13] found that sightings were concentrated outside of the Reserve's boundaries, so a marine refuge was established in the area where vaquita sightings were concentrated [16]. The refuge excludes finfish gillnets and shrimp driftnets, which entangle vaquita, and industrial trawling, which may disrupt vaquita behavior [17], [18]. Currently, direct and indirect economic incentives (Table S1) are coupled with spatial restrictions with the goal of eliminating nets from the entire vaquita distribution area by 2012 (∼8432 km^2^; Figure 1), as specified in the vaquita conservation program [19]. The economic incentives are designed to limit the economic impact of area closures on local fishers [20], [21]. The evolution of fisheries management aimed at reducing vaquita mortality is described in more detail in Rojas-Bracho et al. [22], Bobadilla et al. [23], and Avila-Forcada et al. [21].

The fisheries policies directed at reducing bycatch of vaquita have been designed as single-species management concerned with preventing extinction of the vaquita and reducing the socioeconomic impact of vaquita conservation on the region's fishers [19]. There is a high probability that eliminating nets from the vaquita distribution area would result in an increase in vaquita abundance after 10 yrs, as determined by a single-species population model [24]. Vaquita population trajectories under alternative fisheries policies, obtained using an ecosystem model that incorporated vaquita age structure and diets, were consistent with results of the single-species model [25]. However, calls for fishery management to address broader ecological and conservation goals in the Gulf [26]–[28] and globally [29]–[31] suggest the need to weigh the impacts of fisheries policies on the broader ecosystem.

Fisheries policies directed at reducing vaquita bycatch could have significant ecosystem-level effects for the Upper Gulf of California, because of their spatial extent and their focus on fisheries with high ecological impacts [32], [33]. The Upper Gulf is characterized by high productivity driven by seasonal upwelling and constant tidal mixing [34]. This productivity drives highly profitable artisanal gillnet fisheries, mainly for curvina (*Cynoscion othonopterus*), sharks, skates and rays; driftnet fisheries for blue shrimp (*Litopenaeus stylirostris*); and industrial benthic trawl fisheries for blue and brown shrimp (*Farfantepenaeus aztecus* [32], [35]). Both artisanal gillnets and industrial trawl fisheries have high bycatch rates [36], [37], while trawling has significant physical effects on the seafloor and reduces the diversity of benthic and demersal communities [38].

‘End-to-end’ ecosystem models have proven useful for exploring the implications of fisheries policies on management objectives [39]. For example, Kaplan et al. [40] used an end-to-end model, built in the Atlantis software, to explore the consequences of various gear switching and spatial management scenarios in the California Current. Atlantis models include a coupled dynamic representation of biophysical, ecological, economic and social components of the system, enabling users to explore alternate fisheries management strategies in the context of diverse, interactive ecosystem processes [41].

We used an Atlantis model of the Northern Gulf of California [42], [43] to examine the effects of existing and proposed fisheries policies directed at reducing vaquita bycatch. We compared a reference scenario that did not include any actions for vaquita protection with four scenarios that simulate the current vaquita refuge [17]–[19], [44], planned expansions of the refuge within the species recovery plan [19], and the most recent expert-recommended spatial closure [45]. The scenarios tested combine spatial closures for industrial shrimp trawls, finfish gillnets, and shrimp driftnets while allowing operation of a new artisanal light shrimp trawl that eliminates vaquita bycatch [46]. We examined how the simulated scenarios affected biomass, catch, and diet composition of species of conservation concern and target species. We also analyzed the effects of fisheries policies on performance metrics of ecosystem function and structure; these metrics are intended to reflect changes in ecosystem attributes and can be linked to specific management objectives [47]. The ecosystem metrics included were biodiversity, trophic level of the system, trophic level of catch, system organization, and habitat integrity.

Our overall aim was to illustrate how the indirect consequences of fisheries policies can support conservation objectives, reveal potential tradeoffs, and strengthen long-term management plans. We found indirect effects on species and ecosystem function and structure as a result of vaquita management actions. In general, scenarios that increased ecosystem function and structure resulted in lower economic performance indicators, pointing to the need to consider ecological and economic tradeoffs as part of integrated management.

## Methods

### Atlantis Ecosystem Model

The technical specifications of the Atlantis code base and a review of existing applications are detailed in Fulton et al. [41], [48]. Atlantis is a spatially explicit modeling framework that incorporates multiple submodels that represent oceanography (flux of water, heat and salt), biogeochemistry (primarily N cycling), food web interactions and fisheries. Atlantis is a deterministic model, tracking flows of limiting nutrients through the main biological groups using a system of differential equations solved on a 12-hour time step. Atlantis appears to capture the dynamics observed in real ecosystems and produces spatial zonation and long-term cycles characteristic of natural systems [49]. Aspects of structural uncertainty and parameterization of the Atlantis model are considered by Fulton [50] and Fulton et al. [48], [51], while Link et al. [52] summarize both challenges and future directions for handling uncertainty in ecosystem models. However model limitations are not obstacles to using Atlantis as a strategic model for illustrating broad-scale tradeoffs [49].

Atlantis can be used as a policy exploration tool to predict management policy efficacy for target populations and to understand associated effects on ecosystem components. Importantly, ecosystem models such as Atlantis are meant for strategic evaluations (i.e. ranking policy options) and not for tactical management decisions (i.e. setting management quotas) [41], [53]. Currently, there are 13 Atlantis models being used to support ecosystem-based management, with several others under development [41].

Biotic ecosystem components are typically represented in functional groups: groups of species aggregated according to life history, feeding, or niche similarities. The main model dynamics and processes in Atlantis include two-way trophodynamic coupling, meaning that predators influence prey abundance and vice versa; dynamic weights-at-age; multiple options for describing predator-prey relationships; density dependence arising from both stock-recruit relationships and explicitly modeled resource limitation; and directed movements (i.e. seasonal migrations and foraging) [40]. In existing Atlantis models, target species are represented with sufficient detail to evaluate direct effects of fishing, while other species are aggregated into functional groups with enough resolution to capture human, trophic, and climate impacts on the ecosystem [40], [41], [48], [54]. The model includes a three-dimensional representation of the spatial extent; irregular boxes or polygons represent important bioregional features. Exchange of biomass occurs between polygons based on seasonal migration and foraging behavior, while fluxes of water, heat and salinity across polygon boundaries can be represented by a coupled hydrodynamic model. Subroutines represent biological processes between functional groups, including consumption, production, waste production, recruitment, habitat dependency and mortality, including predation, senescence, and fishery removals; the equations for these processes are described in [48].

### Northern Gulf of California Atlantis model

The Northern Gulf of California model has been applied to test the future ecosystem-level impacts of current fisheries policy and a range of potential policies [25], [43], [55]. Initial model conditions are described in Ainsworth et al. [42]; they represent the ecosystem structure and function for 2008 and provide a detailed representation of the Northern Gulf's oceanography, historical fishing patterns, migration and movement of key species, and variability in diet compositions. The model has been calibrated to fit historical catch series per functional group and tuned through the analysis of catch and biomass equilibria under a range of fishing pressures. The calibration process is iterative since the slow-run time in Atlantis prevents automated estimation of model parameters. Instead, state and rate parameters (i.e. recruitment variables, prey availabilities, predator consumption, mortality, and growth) are adjusted in order to generate realistic system behavior and fit predictions to observations. This overall strategy has been used in all Atlantis models built to date [48], [56]–[58]. A summary of the calibration and tuning process is provided in Text S1.

The model domain extends over 57800 km^2^, from the Colorado River Delta to the northern tip of Baja California Sur (Figure 1). The model area is divided into 66 boxes or polygons. The design of the polygons considered four major factors: the locations of the marine reserves in the region; bathymetry at the 25, 80, 150, 500, and 1,000 m isobaths; the location of fishing ports; and fishery-use areas indicated by Cudney-Bueno and Turk-Boyer [35]. Each polygon includes one sediment layer and up to six water depth layers. The irregular polygons allow the model to capture the critical dynamics of the system while being computationally efficient in homogeneous space. The model is driven by biological, chemical and physical processes that are replicated within each polygon and layer. Fluxes of water, heat, and salt are forced by a Regional Oceanographic Model System (ROMS) that reflects oceanographic conditions in the region from 1985–2008 [34]. Water flux drives the advection of plankton, nutrients and waste cycling; heat affects growth, consumption and primary production rates.

The biological components of the model include 63 functional groups, including 27 fishes, one seabird, 2 sea turtles, 5 mammals, 5 plankton, 18 invertebrates, algae, seagrass, and 2 forms of bacteria (pelagic and benthic), as well as 3 detritus groups: carrion (dead matter, large particles), refractory (cohesive, small particles), and labile (easily disassociated, small particles). The spatial distribution and abundance of each functional group are defined per model polygon and depth layer. The vertebrate groups are age-structured, but invertebrates are modeled as biomass pools. Atlantis tracks abundance and biomass for each pool and age class as mg N·m^−2^. Calculations of predation rates use a Holling Type II functional response; this allows diet composition to vary through time, considering density-dependent effects related to varying abundance of prey items. Feeding rates also vary dynamically according to gape limitation and the state of any prey refuges (for habitat dependent groups). The predation rate is also affected by the spatiotemporal segregation of predator and prey; maximum feeding rates will only occur when the prey and predator coincide in the same polygons and depth layers. Thus, rates of feeding respond to seasonal and diel movement patterns.

### Scenarios

We simulated the impact of five management scenarios. The scenarios began with the same parameterization of ecology and oceanography, such that the differences between scenarios result from the dynamics of fishing. Fishing is simulated on a per-fleet basis; 32 fleets (Table S2) represent our best understanding of the current fishing patterns in the Northern Gulf of California. The fleets are defined based on gear used, targets, bycatch, base ports and fishery utilization areas (see [43]). Each of these fleets has specific fishing areas; we specify the proportion of each model polygon that is open or closed to individual fleets. Fishing mortality is imposed by the fishing fleets onto all relevant functional groups. The scenarios ran over 30 years, from 2008 to 2038. This time period allows the model to reach stable long-term biomass dynamics (quasi-equilibrium), capturing the effect of management scenarios on functional groups with varying life spans.

Initially, we simulated a reference scenario that did not include any management actions for vaquita protection (‘No vaquita management’ scenario). We began simulations for the No vaquita management scenario at 2008 biomass levels. We based initial catches on the average of 2000–2007 catches (Table S3), assembled from official fishery statistics, port-level surveys, and fisher log books [42]. We used data on catch and bycatch composition to assign a proportion of the catch of each functional group to each of the 32 fleets in the model (Table S4; [43]). Vaquita abundance was set at 245, the most recent estimate [14] and vaquita mortality rate was 0.15 year^−1^ (15%), the median estimate for 2007 [24]. This scenario utilizes an estimate of the current degree of compliance with existing fisheries restrictions made by Ainsworth et al. [43] and incorporates a 30% reduction in trawl effort within the Upper Gulf Biosphere Reserve (Figure 1) implemented in 2008 [17], part of a voluntary program to reduce shrimp trawl effort nationwide [59].

The No vaquita management scenario was then compared to four scenarios that simulate management actions directed at eliminating vaquita bycatch [19], [60]. These scenarios (Figure 1) each include a 1264-km^2^ spatial closure to industrial shrimp trawls within the current vaquita refuge [17], [18]. The scenarios then simulate progressively larger spatial closures for shrimp driftnets and finfish gillnets in the area where vaquita sightings are concentrated [13]; they also allow the shrimp driftnet fleet to switch to a light trawl with no vaquita bycatch [61], instead of being excluded. The four scenarios are as follows, in order of increasing restrictions on fisheries:

1. Vaquita refuge scenario: includes a 1264-km^2^ spatial closure [17], [19], representing the 2010 status quo.
2. Extended refuge scenario: includes a 3579-km^2^ spatial closure representing an option from the species recovery plan [19].
3. Primary area scenario: includes a 5339-km^2^ closure that excludes nets from the main vaquita distribution area [14]. This corresponds to a recent recommendation of the International Committee for the Recovery of the Vaquita, an ad-hoc scientific committee charged with making management recommendations to the Mexican government [45].
4. Distribution area: closes off the entire known vaquita range (8432 km^2^). This is equivalent to the 2012 target in the species recovery plan [19].

The closures were simulated as partial or complete spatial closures to the shrimp driftnet and finfish gillnet fleets in the model polygons affected, with fishing mortality reduced proportionally to area closed. The conservation program being implemented to eliminate vaquita bycatch is designed to minimize redistribution of fishing effort. Fishers either receive a payment for conservation (rent-out), replace their gears for vaquita-safe gears (switchout), or are paid to leave the fishery entirely (buyout) (Table S1); thus, we do not consider possible increases in illegal fishing.

In each of these four spatial closure scenarios a new light shrimp trawl fleet that eliminates vaquita bycatch [46] was allowed to operate within the area closed to shrimp driftnets and finfish gillnets. We assumed that as the area closed to these gears increases, adoption of the light trawl will increase, as many fishers want to continue fishing [32] and shrimp are profitable [62]. This is consistent with data showing that fishers that enrolled in the buyout were those close to retirement and that no fishers have opted to leave the fishery since 2010 [21]; we do not consider participation in the rent-out option. To simulate the new light shrimp trawl, we reduced shrimp catch by 13% per unit fishing effort relative to the shrimp driftnet fleet [63] and increased bycatch of species other than vaquita by 11% [63], [64]. Bycatch composition of the light trawl fleet also varied relative to the shrimp driftnet fleet. Dominant groups in the shrimp driftnet include crabs and lobsters, drums and croakers, flatfish, and small demersal fish [64]. In the shrimp light trawl fleet, drums and croakers, flatfish, and small demersal fish also represent a large proportion of bycatch in addition to small reef fish and large pelagics ([63], [64]; Table S5). Fishing mortality of vaquita was set to 0 for this fleet.

### Analysis

We present functional group biomass and catch results only for the fifteen polygons directly affected by management scenarios, rather than the model extent (Figure 1), to better illustrate the effect of fisheries policies directed at reducing vaquita bycatch in the Upper Gulf of California. We focus on species of conservation concern and priority target species as indicated in the Upper Gulf Biosphere Reserve management plan, excluding blue crabs (*Callinectes* spp.) because of unstable behavior in this high-productivity model group (Table S6; [60]). We also examined additional target groups for unexpected responses. As performance metrics for individual groups we include biomass, catch, exploitation rate (catch/biomass), and diet composition. Unless specified, in the Results we present biomass for the end of the simulation (2038) to show long-term trends and we present catch for the end of the first year (2009), to reflect the immediate effect of management scenarios not confounded by long-term biomass trends.

We then determined performance metrics of ecosystem function and structure: biodiversity, trophic level of the system, trophic level of catch, system organization, and habitat integrity. Biodiversity was calculated using the Q-90 statistic [65], which represents the slope of the cumulative species abundance curve and reflects both species evenness and richness. We used the 51 major vertebrate and invertebrate functional groups for the calculations.

Trophic level of the system and trophic level of catch were determined as: 
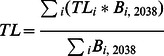
 where TL is trophic level for each functional group *i* (Table S7), *B* is biomass in 2038 for that functional group.

To characterize which scenario had the largest impact on ecosystem organization, we used a reorganization index, modified from Samhouri et al. (2009). This index is calculated as the sum, across all functional groups, of the absolute difference in the relative biomass (B*_i_*/B_Total_) of each functional group (*i*) at the end of the first year (2009) and at end the simulation (2038).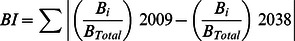.

This index is highest in the scenario where groups exhibited the largest differences in biomass between 2009 and 2038.

Though bottom gears are known to damage habitat [66], [67], quantifying this impact for the dynamic ecosystem model is difficult with available data from the Gulf of California. Instead, for each scenario we calculated a simple index of spatial overlap between gear impacts and habitat [40]. This habitat index estimates the amount of habitat left undisturbed by fishing; for each scenario, the index was calculated based on the relative impacts of particular gear types on substrate [68], substrate per polygon, and fishing effort per gear type and polygon. We assumed that each gear type acted independently on a polygon; therefore the proportion of intact habitat, taking into account the effects of all gears, was the product of the proportion of remaining intact habitat from each gear: 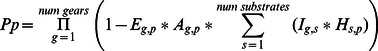 where *Pp* is the proportion of habitat in polygon p that remains intact; *A_g,p_* is the proportion of polygon p open to fishing by gear *g*, *E_g,p_* is the effort by that gear in that polygon, relative to initial levels; *I_g,s_* is the impact factor per gear and substrate [68], and *H_s,p_* is the proportion of the habitat that is substrate *s*. The habitat integrity metric is then: 
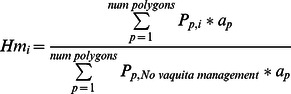
 where the habitat integrity metric (*Hm*) is the undisturbed habitat in scenario *i* relative to the No vaquita management scenario, and *a_p_* is area of each polygon (km^2^). The shrimp light trawl is designed to have less impact than the industrial bottom trawlers [46], but we could not obtain quantitative data on its expected benthic impact; thus we assigned it 50% of the impact factor for bottom trawlers.

We also analyzed economic benefit for artisanal net fleets and other artisanal fleets, since they are directly affected by vaquita management actions. For each scenario, Net benefit per fleet was calculated as the average Net benefit for the last five years of the simulation. For any given year, Net benefit is the sum of the Net benefits (NB) derived from the harvest of all functional groups caught by the fleet: 
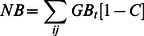
 where GB is gross benefit (i.e. value of catch) for year *t* and *C* is cost rate for fishing. We assumed a cost rate of fishing of 32% for artisanal fleets [32]. Value of catch is dollars tonne^−1^ by functional group (Table S7) for 2010 or the most recent year for which data was available. Catch per fleet are tonnes over the whole area where the fleet can operate.

## Results

Our goal is to evaluate the impacts of the four management strategies in terms of four types of metrics: species of conservation concern, priority target species, ecosystem function and structure, and economic benefit. Responses of vaquita and economics are detailed in Morzaria-Luna et al. [25]. Here, we present a combined discussion of all these axes of management performance, to allow evaluation of the tradeoffs inherent in these policy choices.

### Species of conservation concern

**Figure 2:**
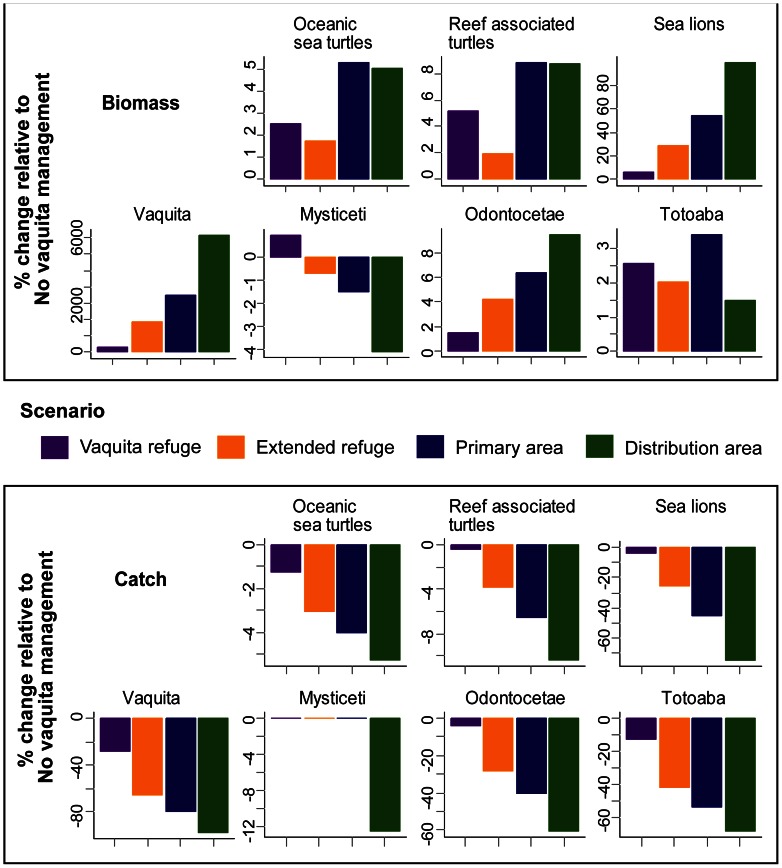
Biomass (top panel) and incidental catch (bottom panel) for species of conservation concern under various management scenarios. Bars show percent change relative to the No vaquita management scenario, relative to 2038 for biomass and to 2009 for catch.

Overall, populations of species of conservation concern increased as the spatial area of closures increased. Generally, the most restrictive spatial closures (Distribution area scenario) resulted in the highest biomass of species of conservation concern in year 2038 relative to the No vaquita management scenario (Figure 2). Biomass of sea lions (*Zalophus californianus californianus*; pinniped functional group) in the Distribution area scenario was 99% higher than in the No vaquita management scenario. Increases in biomass for other species of conservation concern were less than 10%. Here we focus on responses (Figure 2) in the area affected by management actions (blue polygons in Figure 1). Nonetheless, these responses were consistent with results calculated at the scale of the entire model, once spatial distribution of the species is taken into account (Text S2).

**Figure 3:**
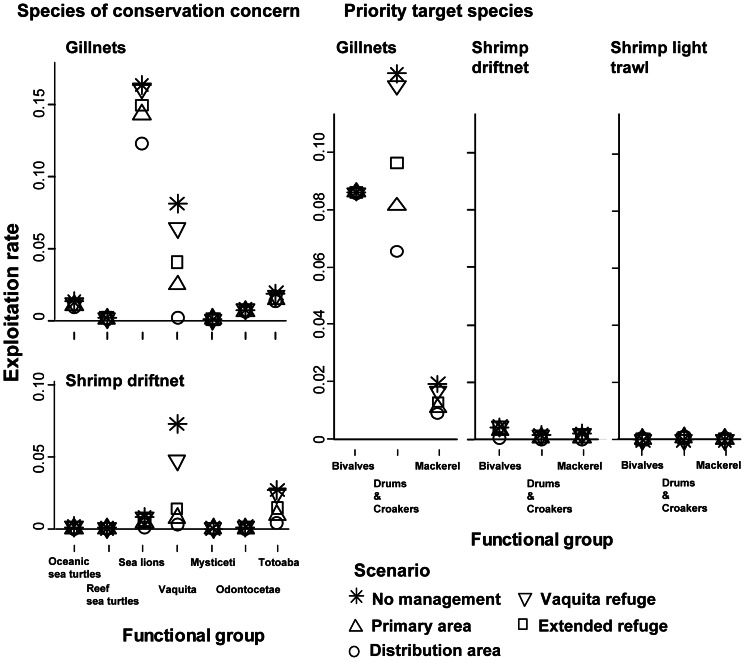
Exploitation rate (catch/biomass) of species of conservation concern and priority target species in the shrimp industrial trawl and shrimp driftnet fleets of the Upper Gulf and the gillnet fleet for the complete model extent. The exploitation rate across scenarios was calculated using catch relative to biomass in 2009.

Incidental catch of groups of conservation concern decreased under highly restrictive management scenarios; these reductions were evident by the first year of the simulations. For example, 2009 bycatch of sea lions, whales and dolphins, and totoaba (*Totoaba macdonaldi*), a threatened endemic sciaenid fish, decreased over 60% in the Distribution area scenario relative to the No vaquita management scenario (Figure 2). Exploitation rate of sea lions, sea turtles, and totoaba decreased by>80% in shrimp driftnets and by at least 20% in finfish gillnet fleets in the Distribution area scenario relative to the No vaquita management scenario (Figure 3). The amount of prey consumed by each group varied; in general, species consumed more prey in scenarios where reduced fishing effort led to subsequent increases in biomass (Figure S1).

### Priority target species

**Figure 4:**
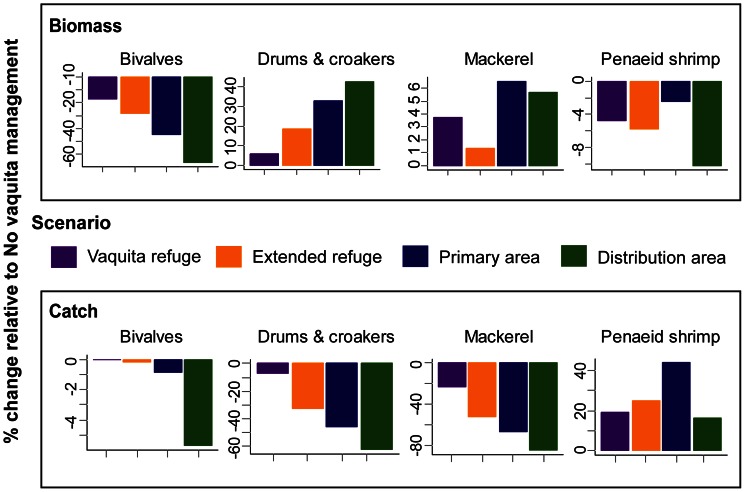
Biomass (top panel) and incidental catch (bottom panel) for priority target species under various management scenarios. Bars show percent change relative to the No vaquita management scenario, relative to 2038 for biomass and to 2009 for catch.

In the case of priority target species, species in higher trophic levels, mackerel (trophic level 3.84) and drums and croakers (TL 3.95), experienced lower catch and subsequently higher biomass in spatial management scenarios relative to the No vaquita management scenario (Figure 4). Catch of mackerel in 2009 declined 84% and catch of drum and croaker declined 62% in the Distribution area scenario relative to the No vaquita management scenario (Figure 4). Both these species groups are primary targets in the finfish gillnet fisheries, and so their catch decreased as the fleet was excluded from the Upper Gulf. Similarly, catch of shrimp increased 16% in the Distribution area scenario relative to the No vaquita management scenario, resulting in a 10% decrease in biomass (Figure 4). Catch of several non-priority target species targeted by finfish gillnets also decreased > 50% in the Distribution area scenario relative to the No vaquita management scenario, including Amarillo snapper (*Lutjanus argentiventris*) (−79%), guitarfish (−57%), large pelagics (−54%), Pacific angel shark (*Squatina californica*) (−83%), and small migratory sharks (−83%), leading to biomass increases by year 30.

The exploitation rate of mackerel and drums and croakers in shrimp driftnets decreased 87% and 85% respectively (Figure 3) in the Distribution area scenario relative to the No vaquita management scenario; at the same time the exploitation rate increased in the shrimp light trawl as the gear was allowed to operate in a larger area. However, the exploitation rate of drums and croakers by the shrimp light trawl in the Distribution area scenario was still 20% lower than in the shrimp driftnet under the No vaquita management scenario. The amount of prey consumed by each group varied; in general, species consumed more prey in the Distribution area scenario (Figure S1; Table S8).

### Ecosystem structure and function metrics

Biodiversity (Kempton's Q-90) showed small increases in management scenarios relative to the No vaquita management scenario (Table 1). The largest value in 2038 (4.26) was achieved in the Distribution area scenario, and the lowest in No vaquita management (4.052). A small decrease in Q-90 represents a large change in the ecosystem because the metric represents the cumulative species abundance curve [65]. Trophic level of the system varied 0.07 units between management scenarios (Table 1). The lowest value in 2038 (3.24) occurred under the No vaquita management scenario and the highest in the Vaquita refuge scenario. Trophic level of catch varied 0.05 units between management scenarios; the lowest value in 2038 (3.58) occurred under the Distribution area scenario and highest (3.63) in the No vaquita management scenario (Table 1); trophic level of catch decreased as total catch decreased. When finfish gillnets and shrimp driftnets were excluded from the Upper Gulf in the Distribution area scenario, catch of all target groups (except shrimp) decreased. From a bioenergetics perspective, the difference in trophic level represented a 3% difference in the primary production necessary to sustain a given amount of catch [69]. The reorganization index was highest in the Extended refuge scenario (1.52), where groups showed the largest response to management restrictions (Table 1). The index was lowest under the No vaquita management scenario (1.38), where biomass of individual functional groups showed the smallest changes throughout the simulation compared to other scenarios. The most restrictive spatial management scenario (Distribution area) resulted in the largest increase in habitat integrity (value of 1.5x No vaquita management scenario). The improvement in habitat integrity was less in other scenarios as the area subject to spatial restrictions was smaller.

**Table 1:** Results for performance metrics of ecosystem function and structure for the management scenarios tested.

Scenario	Biodiversity	Trophic level of system	Trophic level of catch	Reorganization index	Habitat integrity
No vaquita management	4.046	3.24	3.635	1.384	1
Vaquita refuge	4.096	3.31	3.620	1.484	1.090
Extended refuge	4.172	3.31	3.604	1.517	1.285
Primary area	4.218	3.30	3.585	1.445	1.315
Distribution area	4.260	3.30	3.584	1.500	1.516

The habitat integrity metric is scaled relative to the No vaquita management scenario.

### Food web effects

**Figure 5:**
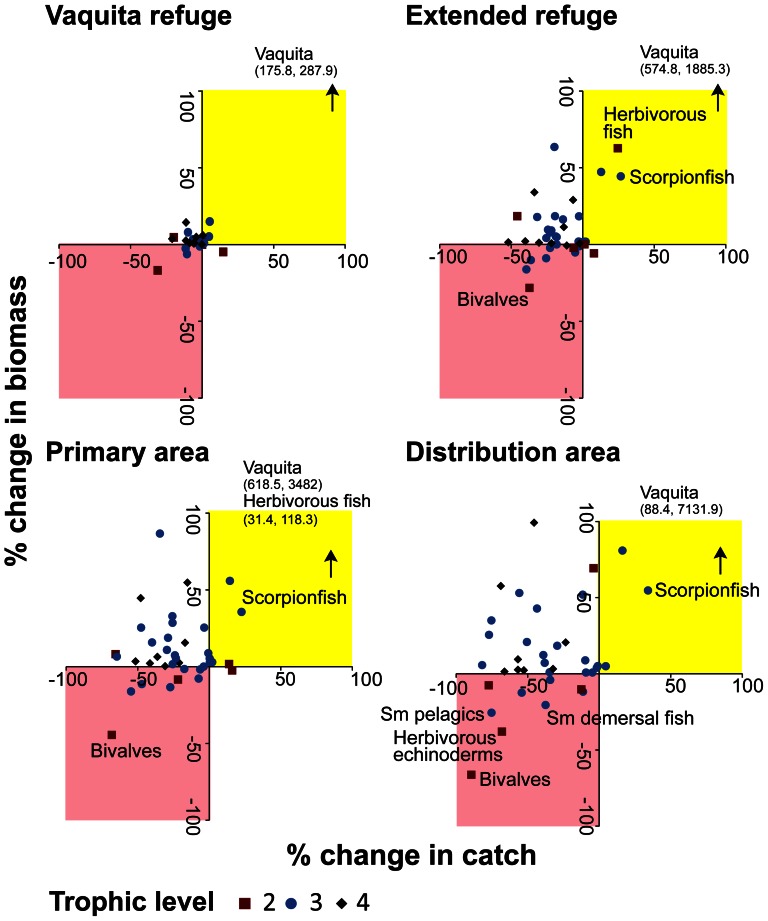
Percent change in catch and biomass for each management scenario relative to the No vaquita management scenario in 2038. Species are binned by trophic level as indicated by the markers. We indicate groups with>±20% change in both catch and biomass. The pink shading indicates species with aa decrease in both catch and biomass. The yellow shading indicates species withan increase in both catch and biomass. The arrows indicate the groups that are off the scale.

We found that the fisheries policies implemented in the management scenarios led to cascading effects throughout the food web by 2038. These complex effects were examined using a combination of catch and biomass ratios relative to the No vaquita management scenario for individual scenarios (Figure 5) and ratios of prey mortality and predator consumption (Table S8). The spatially-restrictive Distribution area and Primary area scenarios resulted in large trophic effects. Most groups in trophic levels 3 and 4 increased in biomass and decreased in catch as fishing mortality decreased; almost all species are either target or bycatch of the shrimp driftnet and finfish gillnet fleets. Groups such as scorpionfish (TL 3.7), skates, rays and sharks (TL 3.3), and Gulf coney (TL 3.4) experienced a release from predation in combination with a reduction in fishing mortality, leading to increases in both catch and biomass relative to the No vaquita management scenario. In response to these biomass increases, prey groups such as bivalves (TL 2), small pelagics (TL 3.1), and small demersal fish (TL 3.8) declined in biomass and catch.

### Economics

The economic cost of vaquita management actions was unequally divided between fishing fleets; the loss of value from finfish gillnet fisheries resulting from spatial restrictions was never recovered. The average annual net benefit (value of catch minus costs) of artisanal net fleets, which include finfish gillnets, shrimp driftnets, and shrimp light trawl, was US$49±0.5 million in the Distribution area scenario by 2038; this represents a US$23 million decrease relative to the No vaquita management scenario. Fishery gains from spatial management are modest because net benefit is driven by abundant finfish (over 65% of net benefit across scenarios) rather than harvest of sedentary species and overfished species more likely to benefit in reality from spatial closures. Overall, functional groups contributed unequally to net benefit dependent on spatial closures. For example, the catch value of Gulf grouper, large pelagics, and drums and croakers decrease relative to the No vaquita management scenario. A greater proportion of catch value under spatial management comes from herbivorous fish and sharks. Other artisanal fleets, including longline, handline, traps, and dive fisheries benefited from spatial management, and average net benefit for these fleets outperformed the No vaquita management scenario by US$ 2–8 million by the end of the simulation. These gains in net benefit result from higher catch of groups that experience decreased fishing pressure in scenarios with spatial closures, including herbivorous fish, sharks, Amarillo snapper, and drums and croakers. A detailed analysis on the effects on vaquita management policies on economic benefit of fisheries catch is found in Morzaria-Luna et al. [25], including consideration of discounting (the current capital value of future income, reflecting uncertainty and lost opportunity costs).

## Discussion

In recent years, there has been a shift toward the implementation of an ecosystem-based approach to fisheries management that recognizes and addresses the indirect effects of fishing [70]; ecosystem-based management can help ameliorate indirect effects by taking into account a variety of ecosystem components (i.e. non-target species, trophic interactions, protected species) [71]. Nonetheless, it is still common for fisheries policies to focus on a single species as if it was an autonomous system rather than being embedded in wider ecological, socioeconomic, and institutional structures and processes [72]. In the Gulf of California, one of the most biodiverse seas in the world and Mexico's chief source of fishery resources for national and international markets [73], single-species management has led to cases of fishery collapse (and subsequent recovery) related to overfishing of stocks including totoaba [74], shrimp [75], sardine [76], and bigeye croaker (*Micropogonias megalops*) [77]; as well as declines in species of conservation concern including vaquita [22], sea lions [78], sea turtles [79], and whales [80].

We found indirect effects on species and ecosystem function and structure for the Upper Gulf of California as a result of vaquita management actions. Our results exemplify the potential for both positive and negative indirect effects of single-species management and point to complex interactions and tradeoffs. For example, vaquita management actions directly benefited other species of conservation concern, sea lions, sea turtles, whales and dolphins, and totoaba. Most importantly, sea lions showed increasing biomass and lower incidental catch as a result of the exclusion of finfish gillnets and shrimp driftnets. This is a meaningful finding because total abundance in the Gulf of this protected species [81] has declined > 20% between 1994 and 2004 [78]. The smaller increases in totoaba and sea turtle biomass are still important, as lower abundance of these groups has been attributed not only to bycatch but also to other processes and stresses unrelated to fishing that operate at distinct temporal and spatial scales. For example, reductions in the flow of the Colorado River, associated loss in spawning and nursery habitats, and environmental variability interacted in the decline of the totoaba population [74], [82]. In the case of sea turtles, habitat loss and egg poaching are major concerns [79].

Some fish groups targeted by commercial fisheries also benefited from vaquita management actions. Particularly, both catch and biomass of skates, rays, and sharks and Gulf coney increased due to a combination of lower predation and reduced fishing mortality in spatially restrictive scenarios. These increases could benefit the multispecies artisanal fisheries in the Northern Gulf of California, since skates, rays, and sharks are an overexploited but important component in the fisheries of the region [83] and Gulf coney is a species with high market value [84].

We also found negative indirect effects of vaquita management actions. The increase in biomass of higher-trophic level groups (TL 3 and 4), including species of conservation concern, resulted in higher predation pressure on lower trophic levels in scenarios with reduced fishing effort. We previously found that increased predation on small pelagics negatively affected the small pelagic industrial purse seine fishery; lower biomass in spatially restrictive scenarios led to a decrease in net benefit (purse seine vessel profits) relative to the No vaquita management scenario [25]. The sardine fishery, a Marine Stewardship Council-certified sustainable fishery, is an important economic driver in the Northern Gulf [85]. The fishery is characterized by extreme variability in landings due to environmental factors and/or food web feedbacks; thus, the indirect effects of vaquita management could further complicate management of the fishery [86].

The improvements in the performance metrics for individual species' and ecosystem function and structure metrics were limited, since management restrictions only exclude finfish gillnets and shrimp driftnets, while allowing other gears to operate. Most significantly, vaquita management policies only exclude industrial shrimp trawlers from the 1264-km^2^ current vaquita refuge [17], [18]. This fleet has high environmental impacts, including high bycatch of juveniles and threatened species [67], changes in the community structure of the benthos [87], physical changes in the sea floor and water column (caused by sediment suspension), and changes in organic and inorganic matter loading [88]. In contrast, a range of studies within existing marine reserves (those that prohibit fishing) have found reserves can maintain a diverse age-structure, and higher stock abundance and reproductive output in a variety of taxa [89].

**Figure 6:**
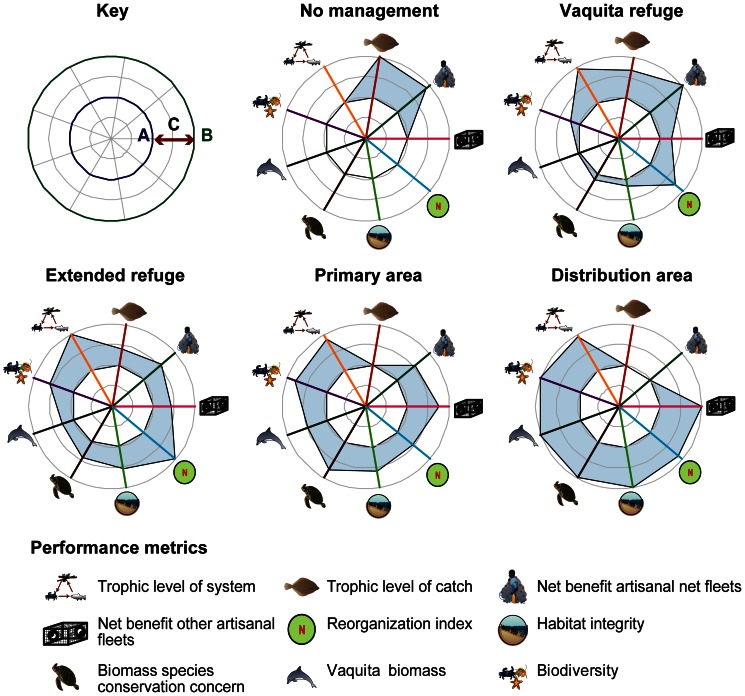
Performance of selected metrics across management scenarios. Since the metrics are not directly comparable (in absolute or relative change), we have scaled the performance metrics between the worst result observed (A/blue circle) and the best result observed (B/green circle); the range in between (C) shows the scope of possible outcomes. Symbols courtesy of IAN/UMCES Symbol and Image Libraries.

To evaluate tradeoffs between scenarios, the performance metrics for species of conservation concern and priority target species and the metrics for ecosystem function and structure can be combined with the vaquita population response and the economic effects for fisheries in the Upper Gulf under alternative fishing policies. Previously [25], we found only the most extensive spatial management scenarios recovered the vaquita population above the threshold necessary to delist the species from Critically Endangered; in the Distribution area scenario, vaquita biomass increased 2.7 times relative to 2008 levels. When all performance metrics are evaluated simultaneously (Figure 6), we find that scenarios that increase ecosystem function and structure result in lower economic indicators. The No vaquita management scenario and Vaquita refuge scenario have high catch of priority target species, shrimp catch, and trophic level of catch but lower performance on other ecosystem function and structure metrics, biomass of species of conservation concern, and vaquita biomass. The rank order of the results was consistent; the Distribution area scenario resulted in the highest ecosystem function and structure metrics, except for trophic level and reorganization index. The Primary area scenario could be a more tenable management goal than eliminating shrimp driftnets and finfish gillnets from the complete vaquita distribution area. This option could provide ecological benefits while representing a compromise between vaquita conservation and fisheries, where vaquita bycatch is low, higher ecosystem function and structure metrics relative to the No vaquita management scenario, and there is a modest decrease in net benefit of fisheries [25].

Thus, preventing extinction of vaquita would require eliminating fishing nets from its distribution area or known range; this would be a process with high economic costs for the fishing community [21], [25]. Economic incentives within the vaquita conservation plan [19] are designed to eliminate net fisheries through payments for conservation, subsidies to accelerate adoption of vaquita-safe technologies, or compensations for fishers to permanently exit the fishery [21]. These incentives are thus designed to reduce fishing effort overall, which could carry high cultural and social costs as fishers might not want or be able to shift to alternate economic activities [21], [32]; limiting the effectiveness of vaquita management actions.

The cumulative impacts of vaquita management actions and other stressors, including those derived from climate and global change, could lead to surprising outcomes not considered here. Climate change could have direct effects on marine food webs, for example causing increases in species mortality, or indirect effects through predator–prey interactions [90]. The effects of climate change on commercially important species could be comparable to the ones produced by fishing [91]. For example, in the Gulf of California environmental variability is dominated by the interaction of decadal and multidecadal events derived from the El Niño–Southern Oscillation (ENSO) and Pacific Decadal Oscillation (PDO); changes in the frequency and severity of these events could lead to ecosystem reorganization [34].

End-to-end models, such as Atlantis, are best suited for strategic analyses such as the one presented here, where the questions involve the interaction of multiple species, biophysical processes, fleets, and management options [41]. Since no model can fully represent the dynamics and behavior of a natural ecosystem, there will always be factors that are not addressed [92]. In our case, these include the effects of simulated scenarios on larval dispersal and connectivity, which could have important management implications as ocean currents transport fish and invertebrate larvae from the northern to the southern Gulf during winter [93]. We used aggregated functional groups rather than species for some important commercial target species (i.e. Gulf corvina, *Cynoscion othonopterus*) which could confound the effects of particular gear restrictions. Importantly, we did not consider displacement of fishing effort nor economically-driven changes in effort, which could oversimplify fishers' response to management actions [94].

This is the first analysis of the effects of alternate vaquita policies on other species of conservation concern, target species, and ecosystem-level effects. Previous analyses of vaquita management actions have focused on vaquita population dynamics [24], [95] and the socioeconomic impact on fishers and fisheries in the Upper Gulf of California [21], [32], [96], [97]. Our findings illustrate the need for integrated management that reduces conflicts and simultaneously achieves conservation, ecological, and socioeconomic objectives. Extensive work would be needed to implement ecosystem-based management in the Upper Gulf of California, most importantly defining a desired ecosystem state that takes into account the needs and concerns of all stakeholders and aspects of the ecosystem and considers uncertainty derived from stochastic factors including climate change [98], [99]. There is a clear need for an integrated perspective that regulates other activities in the Upper Gulf in addition to commercial fishing (i.e. conservation, aquaculture, sport fishing, tourism) [100]–[102]. Already, Mexican legislation specifies tools (i.e. Marine Protected Areas, ‘ecological ordinance plans’) that are science-based and can coordinate environmental conservation and fisheries management [73], [103]. Given the potential ecological and economic tradeoffs resulting from conservation and management of vaquita, consideration of all possible fisheries and environmental impacts is urgently needed.

## Supporting Information

Figure S1 Prey consumed by species of conservation concern and priority target species, at the end of the 30 yr simulation. Heat map of normalized values for each functional group (across rows), such that the color gradient from yellow to red represents a linear increase between the minimum and the maximum amount of prey consumed across management scenarios.
(TIF)

Table S1 Direct economic incentives that support phasing-out finfish gillnets and shrimp driftnets from the vaquita distribution area. Incentives are for small-scale fishers and fishing cooperatives [1] in the Upper Gulf of California, Mexico. Payments and guidelines for fiscal year 2011 [2].
(DOCX)

Table S2 Fishery fleets for Atlantis model. Modified from Ainsworth et al. [1]. Includes number of functional groups targeted (out of 63) in the model, including target and bycatch groups. See Ainsworth et al. [1] for more information on fleets and functional groups used in the Atlantis model.
(DOCX)

Table S3 Catch by functional group used as baseline in the No management scenario. Catches are the average of the 2000–2007 model catch series from Ainsworth et al. [1] summed for all fleets. Vaquita mortality rate was set at 0.15 year-1(15%), the median estimate prior to 2007 [2]. Since the publication of Ainsworth et al. [1], the model has been simplified to only include catch for a generic Penaeid shrimp group rather than for separate shrimp groups. Ainsworth et al. [3] provide species composition for each functional group.
(DOCX)

Table S4 Catch per fleet used as a baseline. Asterisk indicates group is bycatch; fleet name is in bold. Vaquita catch was updated considering a mortality rate of 0.15 year-1 (15%). Bycatch composition for the Upper Gulf shrimp driftnet fleet was updated using recent monitoring data [12]. Otherwise catch per fleet values are unmodified from Ainsworth et al. [3], [10].
(DOCX)

Table S5 Catch for the shrimp light trawl fleet. Bycatch composition was based on INAPESCA & NMFS [1] and considering a reduction in shrimp catch of 10% [1] and an increase in the ratio of shrimp to bycatch (other than vaquita) of 11% [1], [2]. Vaquita bycatch was set to 0 for this fleet.
(DOCX)

Table S6 Species of conservation concern and priority target species in the Upper Gulf of California and Colorado River Delta Biosphere Reserve management plan [1]. Atlantis functional groups that contain the species are indicated. Note that the functional group Drums and croakers includes both priority target species and species of conservation concern.
(DOCX)

Table S7 Trophic levels and value for each functional group. Trophic level are from Lozano [1] and Froese and Pauly [2]. Values are dollars tonne-1 for 2010 or the most recent year for which data was available. For Penaeid shrimp, prices were set by fleet, weighted by the amount of blue, brown and Japanese shrimp caught. Value information from National statistics for Sonora and Baja California, (Anuarios Estadísticos www.inegi.org.mx), state statistics for Sonora (www.oeidrus-sonora.gob.mx/), and port-level data for both states (unpublished data, A. Cinti, The University of Arizona, acinti@email.arizona.edu); data in Mexican pesos was converted to dollars using the exchange rate from 2005–2010 (www.x-rates.com). † Grouped as cabrilla in statistics. ‡ Grouped as sharks in statistics.
(DOCX)

Table S8 Ratios of prey mortality (prey) and predator consumption (pred) for each functional group across management scenarios relative to the No-management scenario.
(DOCX)

Text S1 Northern Gulf of California model tuning and diagnostics. Excerpted from Ainsworth et al. [3].
(DOCX)

Text S2 Model-wide responses of biomass for species of conservation concern.
(DOCX)
