# Supplementary figures and images for: Indirect Effects of Conservation Policies on the Coupled Human-Natural Ecosystem of the Upper Gulf of California
Source: PLoS One. 2013 May 15;8(5):e64085. doi: 10.1371/journal.pone.0064085 (PMC3654961; doi:10.1371/journal.pone.0064085)

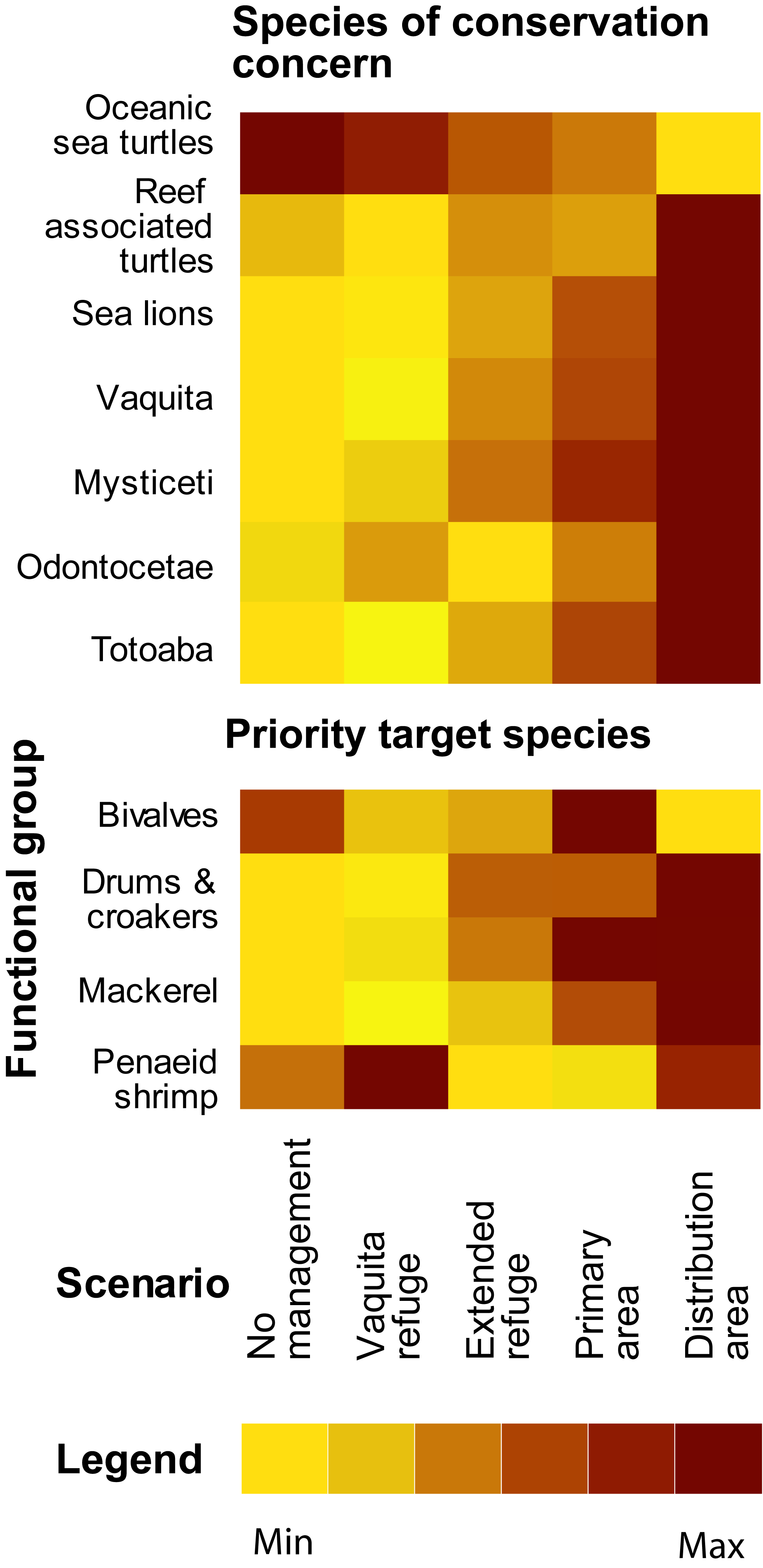

Supplement: Figure S1 — Prey consumed by species of conservation concern and priority target species, at the end of the 30 yr simulation. Heat map of normalized values for each functional group (across rows), such that the color gradient from yellow to red represents a linear increase between the minimum and the maximum amount of prey consumed across management scenarios. (TIF) [file pone.0064085.s001.tif]
