# Supplementary material for: Indirect Effects of Conservation Policies on the Coupled Human-Natural Ecosystem of the Upper Gulf of California
Source: PLoS One. 2013 May 15;8(5):e64085. doi: 10.1371/journal.pone.0064085 (PMC3654961; doi:10.1371/journal.pone.0064085)
Supplement: Table S1 — Direct economic incentives that support phasing-out finfish gillnets and shrimp driftnets from the vaquita distribution area. Incentives are for small-scale fishers and fishing cooperatives [1] in the Upper Gulf of California, Mexico. Payments and guidelines for fiscal year 2011 [2]. (DOCX) [file pone.0064085.s002.docx]

| Option |  | Requirements | Benefits |
| --- | --- | --- | --- |
| Rentout | Annual payment to stop fishing | Agree to stop fishing in the Vaquita refuge | ~ $ 3, 500 USD annually |
| Switchout | Substitution of gillnets and driftnets for gears with no vaquita bycatch | Turn in nets. Agree to use alternative gear. | ~ $ 25, 000 USD, one-time payment. New fishing permit. |
| Buyout | Fishermen stop fishing and are given a payment destined specifically for an alternate business or economic activity | Turn in boat, nets and permits | ~$ 25 - 35, 000 USD, one-time payment (depending on number of permits) |

1. SEMARNAT (2008) Programa de Acción para la Conservación de la Especie: Vaquita (*Phocoena sinus*). Estrategia integral para el manejo sustentable de los recursos marinos y costeros del Alto Golfo de California. Mexico, D.F.: Secretaría del Medio Ambiente y Recursos Naturales. 106 p. Available:http://www.conanp.gob.mx/pdf_especies/PACEvaquita.pdf.

2. SEMARNAT, CONANP (2010) Lineamientos para el otorgamiento de apoyos del Programa de Acción para la Conservación de la Especie: Vaquita (*Phocoena sinus*). Ejercicio fiscal 2010. Available:http://www.conanp.gob.mx/contenido/pdf/LINEAMIENTOS%20PACE%20VAQUIITA%2020100001__.pdf.
