# Supplementary material for: Indirect Effects of Conservation Policies on the Coupled Human-Natural Ecosystem of the Upper Gulf of California
Source: PLoS One. 2013 May 15;8(5):e64085. doi: 10.1371/journal.pone.0064085 (PMC3654961; doi:10.1371/journal.pone.0064085)
Supplement: Table S2 — Fishery fleets for Atlantis model. Modified from Ainsworth et al. [1]. Includes number of functional groups targeted (out of 63) in the model, including target and bycatch groups. See Ainsworth et al. [1] for more information on fleets and functional groups used in the Atlantis model. (DOCX) [file pone.0064085.s003.docx]

| **#** | **Fleet name** | **Ports** | **# target groups** | **Gear description** | **Main groups targeted** |
| --- | --- | --- | --- | --- | --- |
| 1 | Industrial shrimp trawl | Guaymas | 39 | Trawl net | Shrimp |
| 2 | Industrial shrimp trawl | Puerto Peñasco | 39 | Trawl net | Shrimp |
| 3 | Shrimp driftnet | Desemboque, Golfo de Santa Clara, Puerto Peñasco, San Felipe, San Jorge, San Luis | 7 | Gill net | Shrimp |
| 4 | Shrimp driftnet | Bahia Kino | 6 | Gill net | Shrimp |
| 5 | Finfish trawl | All | 12 | Trawl net | Finfish |
| 6 | Offshore demersal gillnet | All | 24 | Gillnet | Small migratory sharks |
| 7 | Inshore demersal shark fleet | All | 5 | Longline and gillnet | Sharks, Angel Shark, migratory Sharks, guitarfish |
| 8 | Inshore pelagic shark fleet | All | 1 | Longline and gillnet | Large pelagic sharks |
| 9 | Inshore demersal gillnet | All | 16 | Gillnet | Guitarfish, skates, flatfish, scorpionfish |
| 10 | Inshore gillnet Curvina | Golfo de Santa Clara, Puerto Peñasco, San Felipe | 10 | Gillnet | Curvina golfina *Cynoscion othonopterus* |
| 11 | Inshore gillnet other | All | 13 | Gillnet | Mullets, Sea turtles |
| 12 | Offshore pelagic gillnet | All | 13 | Gillnet | Sierra, Jacks |
| 13 | Industrial pelagic longline (medium boats) | All | 4 | Longline | Sharks, tuna, jacks & finfish |
| 14 | Longlines (pangas, demersal) | All | 14 | Longline | Gulf coney, extranjero, mustelids |
| 15 | Longlines (pangas, pelagic) | All | 7 | Longline | Sharks, rays, drums, croakers, groupers, snappers |
| 16 | Demersal handline | All | 17 | Handline | Lutjanids, Serranids, Hemulidae, triggerfish |
| 17 | Pelagic handline | All | 13 | Handline | Scombridae, Carrangidae |
| 18 | Blue crab traps | Bahia Kino, Desemboque Seris, Punta Chueca | 1 | Traps | Blue crab |
| 19 | Blue crab traps | Ports North of Desemboque | 1 | Traps | Blue crab |
| 20 | Octopus traps | All | 1 | Traps | Octopus |
| 21 | Fish and lobster traps | All | 9 | Traps | Extranjero, groupers and snappers, large reef fish |
| 22 | Compressor diving | All | 13 | Hooks, harpoons, manual | Clams, oysters, cucumbers, snails, lobster, sea turtles |
| 23 | Octopus compressor diving | All | 1 | Hooks, chemical | Octopus |
| 24 | Penshell compressor diving | All | 1 | Manual | Penshells |
| 25 | Sea cucumber compressor diving | All | 1 | Manual | Sea cucumber |
| 26 | Geoduck compressor diving | All | 1 | Pressure hose | Infaunal / epifaunal meiobenthos, scallops and penshells, carnivorous macrobenthos, bivalves |
| 27 | Jellyfish hand net | All | 1 | Handnet | Jellyfish |
| 28 | Recreational fishing | All | 14 | Fishing pole | Large pelagic, medium pelagic, reef fish |
| 29 | Tuna purse seine | All | 6 | Purse seine | Tuna |
| 30 | Small pelagic purse seine | All | 4 | Purse seine | Small pelagic fish |
| 31 | Macroalgae | All | 1 | Manual | Macroalgae |
| 32 | Other gears | All | 9 | Cast net, harpoons, beach seine, dynamite | Finfish |

1. Ainsworth CH, Morzaria-Luna H, Kaplan IC, Levin PS, Fulton EA (2012) Full compliance with harvest regulations yields ecological benefits: Northern Gulf of California case study. J Appl Ecol 49: 63–72. doi:10.1111/j.1365-2664.2011.02064.x.
