# Supplementary material for: Indirect Effects of Conservation Policies on the Coupled Human-Natural Ecosystem of the Upper Gulf of California
Source: PLoS One. 2013 May 15;8(5):e64085. doi: 10.1371/journal.pone.0064085 (PMC3654961; doi:10.1371/journal.pone.0064085)
Supplement: Table S6 — Species of conservation concern and priority target species in the Upper Gulf of California and Colorado River Delta Biosphere Reserve management plan [1]. Atlantis functional groups that contain the species are indicated. Note that the functional group Drums and croakers includes both priority target species and species of conservation concern. (DOCX) [file pone.0064085.s007.docx]

| **Species of conservation concern** | |  |  |
| --- | --- | --- | --- |
| **Species** | | | **Atlantis functional groups** |
| Vaquita, *Phocoena sinus* | | | Vaquita |
| Corvina golfina, *Cynoscion othonopterus* | | | Drums and croakers |
| Totoaba, *Totoaba macdonaldi* | | | Totoaba |
| Sea turtles: Leatherback, *Dermochelys coriacea*; Olive ridley, *Lepidochelys olivacea*; Loggerhead, *Caretta caretta*; Green, *Chelonia myas agassizi* | | | Oceanic sea turtles, Reef associated sea turtles |
| Sea lions: California sea lion, *Zalophus californianus* | | | Pinnipeds |
| Whales and dolphins: Minke whale, *Baleanoptera acutorostrata*; Blue whale, *Baleanoptera musculus*; Fin whale, *Baleanoptera physalus*; Grey whale, *Eschrichtius robustus*; Pilot whale,  *Globicephala macrorhynchus*; Pygmy sperm whale, *Kogia breviceps*; Humpback whale,  *Megaptera novaeangliae*; Pygmy beaked whale, *Mesoplodon* sp.; Orca, *Orcinus orca*; Sperm whale, *Physeter catodon*; Cuvier’s beaked whale, *Ziphius cavirostris*; Long-beaked common dolphin, *Delphinus capensis*; Risso’s dolphin, *Grampus griseus*; Rough-toothed dolphin, *Steno bredanensis*; Bottlenose dolphin, *Tursiops truncatus* | | | Orca, Mysticeti, Odontocetae |
| **Priority target species** | | |  |
| **Fishery** | **Target species** | | **Atlantis functional groups** |
| Shrimp | Blue shrimp, *Litopenaeus stylirostris*; brown shrimp, *Farfantepenaeus californiensis*; white shrimp, *F. vannamei*; rock shrimp, *Sicyonia* spp. | | Adult blue shrimp, Penaeid shrimp |
| Chano | Gulf croaker, *Micropogonias megalops* | | Drums and croakers |
| Mackerel | *Scomberomorus concolor* | | Mackerel |
| Corvina golfina | *Cynoscion othonopterus* | | Drums and croakers |
| Blue crab | *Callinectes bellicosus, Callinectes arcuatus* | | Adult blue crab |
| Clams | Pacific calico scallop, *Argopecten ventricosus*;*Dosinia dunkeri*; *Chione fluctifraga* and *Ch.californiensis* | | Bivalves |

1. CONANP (2007) Programa de conservación y manejo de la Reserva de la Biosfera Alto Golfo de California y Delta del Río Colorado. México, D.F.: Comisión Nacional de Areas Naturales Protegidas. 319 p.
