# Supplementary material for: Indirect Effects of Conservation Policies on the Coupled Human-Natural Ecosystem of the Upper Gulf of California
Source: PLoS One. 2013 May 15;8(5):e64085. doi: 10.1371/journal.pone.0064085 (PMC3654961; doi:10.1371/journal.pone.0064085)
Supplement: Table S7 — Trophic levels and value for each functional group. Trophic level are from Lozano [1] and Froese and Pauly [2]. Values are dollars tonne-1 for 2010 or the most recent year for which data was available. For Penaeid shrimp, prices were set by fleet, weighted by the amount of blue, brown and Japanese shrimp caught. Value information from National statistics for Sonora and Baja California, (Anuarios Estadísticos www.inegi.org.mx), state statistics for Sonora (www.oeidrus-sonora.gob.mx/), and port-level data for both states (unpublished data, A. Cinti, The University of Arizona, acinti@email.arizona.edu); data in Mexican pesos was converted to dollars using the exchange rate from 2005–2010 (www.x-rates.com). † Grouped as cabrilla in statistics. ‡ Grouped as sharks in statistics. (DOCX) [file pone.0064085.s008.docx]

| **Functional group** | **Trophic level** | **Values in dollars tonne^-1^** | **Year (2010 unless specified)** |
| --- | --- | --- | --- |
| Infaunal epifaunal meiobenthos | 2.0 | 17141.81 | 2008 |
| Scallops and penshells | 2.0 | 16519.55 |  |
| Crabs and lobsters | 3.3 | 12586.32 |  |
| Penaeid shrimp | 2.3 |  |  |
| Shrimp driftnet Upper Gulf |  | 9265.25 |  |
| Shrimp driftnet Kino |  | 9265.25 |  |
| Other gears |  | 9265.25 |  |
| Industrial shrimp trawl Peñasco |  | 7292.72 |  |
| Industrial shrimp trawl Guaymas |  | 6891.71 |  |
| Herbivorous echinoderms | 2.0 | 4618.70 | 2008 |
| Carnivorous macrobenthos | 2.2 | 2828.46 |  |
| Amarillo snapper^†^ | 3.4 | 2740.69 |  |
| Groupers and snappers^†^ | 3.7 | 2740.69 |  |
| Hake | 3.9 | 2689.58 | 2008 |
| Gulf coney | 3.4 | 2073.14 |  |
| Barred pargo | 3.7 | 2005.95 |  |
| Extranjero | 3.4 | 1907.61 | 2008 |
| Sea cucumbers | 2.5 | 1807.03 | 2008 |
| Pacific Angel shark | 4.1 | 1605.21 | 2008 |
| Gulf grouper | 3.4 | 1601.49 | 2007 |
| Small reef fish | 3.3 | 1599.43 | 2008 |
| Flatfish | 4.5 | 1491.33 |  |
| Large reef fish | 3.8 | 1455.46 |  |
| Adult blue crab | 2.2 | 1413.36 |  |
| Bivalves | 2.0 | 1363.41 |  |
| Large pelagics | 4.0 | 1282.57 | 2008 |
| Small migratory sharks^‡^ | 3.7 | 1218.45 |  |
| Large pelagic sharks^‡^ | 4.4 | 1218.45 |  |
| Scorpionfish | 3.7 | 1162.47 | 2006 |
| Skates, rays and sharks | 3.3 | 960.93 |  |
| Snails | 2.5 | 895.78 |  |
| Small demersal fish | 3.8 | 796.01 |  |
| Guitarfish | 3.6 | 743.62 |  |
| Drums and croakers | 4.0 | 685.40 |  |
| Mojarra | 3.5 | 673.89 |  |
| Mackerel | 3.8 | 484.88 | 2009 |
| Herbivorous fish | 2.2 | 471.87 |  |
| Macroalgae | 1.0 | 449.77 | 2008 |
| Squid | 3.2 | 350.21 |  |
| Jellyfish | 3.1 | 78.66 |  |
| Small pelagics | 3.1 | 49.15 |  |

1. Lozano H (2006) Historical ecosystem modelling of the Upper Gulf of California (Mexico): Following 50 years of change. [PhD dissertation]. Vancouver, British Columbia: The University of British Columbia, The Faculty of Graduate Studies. 266 p.

2. Froese R, Pauly D, editors (2009) FishBase. Available: [www.fishbase.org](http://www.fishbase.org). Accessed 2012 Dec 12
