# Supplementary material for: Indirect Effects of Conservation Policies on the Coupled Human-Natural Ecosystem of the Upper Gulf of California
Source: PLoS One. 2013 May 15;8(5):e64085. doi: 10.1371/journal.pone.0064085 (PMC3654961; doi:10.1371/journal.pone.0064085)
Supplement: Table S8 — Ratios of prey mortality (prey) and predator consumption (pred) for each functional group across management scenarios relative to the No-management scenario. (DOCX) [file pone.0064085.s009.docx]

|  | **Trophic level** | **Vaquita refuge** | | **Extended refuge** | | **Primary area** | | **Distribution area** | |
| --- | --- | --- | --- | --- | --- | --- | --- | --- | --- |
|  |  | **Prey** | **Pred** | **Prey** | **Pred** | **Prey** | **Pred** | **Prey** | **Pred** |
| Benthic bacteria | 1.00 | 0.91 | 0.00 | 0.92 | 0.00 | 0.89 | 0.00 | 0.94 | 0.00 |
| Large phytoplankton | 1.00 | 1.00 | 0.00 | 0.95 | 0.00 | 0.93 | 0.00 | 1.06 | 0.00 |
| Macroalgae | 1.00 | 0.95 | 0.00 | 1.00 | 0.00 | 0.94 | 0.00 | 0.96 | 0.00 |
| Microphytobenthos | 1.00 | 0.99 | 0.00 | 1.00 | 0.00 | 1.53 | 0.00 | 1.19 | 0.00 |
| Pelagic bacteria | 1.00 | 0.90 | 0.00 | 0.95 | 0.00 | 1.18 | 0.00 | 0.91 | 0.00 |
| Seagrass | 1.00 | 0.96 | 0.00 | 1.11 | 0.00 | 1.13 | 0.00 | 1.42 | 0.00 |
| Small phytoplankton | 1.00 | 1.25 | 0.00 | 2.06 | 0.00 | 1.25 | 0.00 | 1.24 | 0.00 |
| Bivalves | 2.00 | 1.04 | 0.90 | 1.06 | 0.91 | 1.13 | 1.09 | 1.14 | 0.85 |
| Herbivorous echinoderms | 2.00 | 1.02 | 0.83 | 1.01 | 1.03 | 1.01 | 0.79 | 1.00 | 0.96 |
| Infaunal Epifaunal Meiobenthos | 2.00 | 1.07 | 1.59 | 1.07 | 1.46 | 1.16 | 1.63 | 1.23 | 1.62 |
| Scallops and penshells | 2.00 | 1.67 | 1.21 | 1.53 | 1.15 | 1.71 | 0.93 | 1.69 | 0.96 |
| Sessile invertebrates | 2.00 | 0.90 | 1.37 | 0.88 | 0.82 | 0.89 | 1.33 | 0.98 | 1.03 |
| Small zooplankton | 2.10 | 3.04 | 1.30 | 0.10 | 2.04 | 1.25 | 1.27 | 0.82 | 1.25 |
| Herbivorous fish | 2.19 | 1.03 | 1.23 | 0.79 | 2.07 | 0.75 | 3.26 | 0.56 | 6.75 |
| Adult blue crab | 2.20 | 1.10 | 1.02 | 1.16 | 1.42 | 1.01 | 1.42 | 1.32 | 1.85 |
| Carnivorous macrobenthos | 2.20 | 0.87 | 1.04 | 0.87 | 1.04 | 0.87 | 1.07 | 0.88 | 1.10 |
| Penaeid shrimp | 2.30 | 0.89 | 1.14 | 0.80 | 0.89 | 0.88 | 0.92 | 0.75 | 1.08 |
| Large zooplankton | 2.40 | 0.25 | 3.00 | 0.97 | 0.43 | 0.64 | 1.40 | 0.90 | 0.95 |
| Sea cucumbers | 2.50 | 0.99 | 0.98 | 1.08 | 1.05 | 1.42 | 1.12 | 1.30 | 1.07 |
| Snails | 2.50 | 0.95 | 0.92 | 0.93 | 0.95 | 0.90 | 0.98 | 0.97 | 1.00 |
| Adult blue shrimp | 3.00 | 0.00 | 0.00 | 0.00 | 0.00 | 0.00 | 0.00 | 0.00 | 0.00 |
| Jellyfish | 3.10 | 1.19 | 1.68 | 1.11 | 0.62 | 1.21 | 1.12 | 1.08 | 0.86 |
| Small pelagics | 3.10 | 1.16 | 1.11 | 1.48 | 1.23 | 1.92 | 1.41 | 2.54 | 1.60 |
| Squid | 3.20 | 1.03 | 0.31 | 1.01 | 0.97 | 1.10 | 0.68 | 1.04 | 0.90 |
| Mysticeti | 3.23 | 0.00 | 1.06 | 0.00 | 1.15 | 0.00 | 1.26 | 0.00 | 1.36 |
| Small reef fish | 3.26 | 1.01 | 1.17 | 1.02 | 1.24 | 1.02 | 1.34 | 1.03 | 1.50 |
| Reef associated turtles | 3.27 | 1.03 | 1.00 | 1.05 | 1.00 | 1.08 | 1.00 | 1.10 | 1.01 |
| Skates, rays and sharks | 3.28 | 0.88 | 1.10 | 0.72 | 1.35 | 0.70 | 1.38 | 0.63 | 1.52 |
| Crabs and lobsters | 3.30 | 0.98 | 1.03 | 0.95 | 1.02 | 0.98 | 1.11 | 1.01 | 1.12 |
| Amarillo snapper | 3.40 | 0.92 | 0.97 | 0.83 | 0.94 | 0.79 | 0.92 | 0.74 | 0.87 |
| Extranjero | 3.40 | 1.06 | 0.96 | 1.07 | 0.92 | 0.97 | 0.91 | 0.75 | 0.86 |
| Gulf coney | 3.40 | 1.15 | 0.95 | 1.19 | 0.91 | 1.24 | 0.88 | 1.22 | 0.83 |
| Gulf grouper | 3.40 | 0.99 | 0.94 | 0.97 | 0.71 | 0.94 | 0.67 | 0.95 | 0.56 |
| Leopard grouper | 3.40 | 1.01 | 0.99 | 1.03 | 0.75 | 1.06 | 0.63 | 1.10 | 0.49 |
| Oceanic sea turtles | 3.44 | 1.00 | 0.98 | 0.98 | 0.95 | 0.97 | 0.93 | 0.97 | 0.89 |
| Mojarra | 3.47 | 0.99 | 1.02 | 0.75 | 1.19 | 0.64 | 1.30 | 0.51 | 1.53 |
| Guitarfish | 3.56 | 0.95 | 1.04 | 1.02 | 1.18 | 0.97 | 1.31 | 1.02 | 1.53 |
| Sea birds | 3.60 | 0.98 | 1.11 | 0.79 | 1.45 | 0.67 | 1.86 | 0.51 | 2.59 |
| Scorpionfish | 3.65 | 0.97 | 1.15 | 0.92 | 1.64 | 0.96 | 1.64 | 0.94 | 2.13 |
| Groupers and snappers | 3.65 | 1.02 | 0.98 | 1.01 | 0.74 | 0.97 | 0.58 | 0.91 | 0.48 |
| Small migratory sharks | 3.66 | 1.01 | 1.15 | 1.04 | 1.39 | 1.06 | 1.52 | 1.08 | 1.65 |
| Barred pargo | 3.68 | 0.98 | 1.07 | 0.95 | 1.19 | 0.89 | 1.32 | 0.88 | 1.38 |
| Large reef fish | 3.77 | 0.98 | 1.04 | 0.97 | 1.04 | 1.02 | 0.96 | 1.02 | 0.78 |
| Grunts | 3.79 | 0.99 | 1.13 | 0.86 | 1.53 | 0.82 | 1.97 | 0.71 | 3.01 |
| Small demersal fish | 3.81 | 1.02 | 1.01 | 1.17 | 0.61 | 1.27 | 0.45 | 1.47 | 0.29 |
| Mackerel | 3.84 | 0.95 | 1.01 | 0.98 | 1.03 | 0.95 | 1.08 | 0.98 | 1.15 |
| Hake | 3.90 | 1.01 | 1.51 | 1.03 | 0.73 | 1.05 | 1.01 | 1.07 | 0.96 |
| Drums and croakers | 3.96 | 0.97 | 1.05 | 0.90 | 1.17 | 0.86 | 1.43 | 0.83 | 1.43 |
| Pinnipeds | 4.00 | 1.01 | 1.01 | 1.00 | 1.18 | 0.98 | 1.36 | 0.96 | 1.65 |
| Large pelagics | 4.05 | 0.99 | 1.00 | 0.93 | 0.83 | 0.90 | 0.75 | 0.88 | 0.63 |
| Vaquita | 4.10 | 1.04 | 3.91 | 1.13 | 16.22 | 1.18 | 26.14 | 1.24 | 44.15 |
| Pacific Angel shark | 4.14 | 0.96 | 1.06 | 0.92 | 1.14 | 0.93 | 1.19 | 0.93 | 1.20 |
| Totoaba | 4.20 | 1.00 | 1.01 | 0.98 | 1.05 | 0.97 | 1.11 | 0.96 | 1.19 |
| Large pelagic sharks | 4.37 | 1.03 | 1.01 | 1.09 | 0.99 | 1.13 | 0.97 | 1.18 | 0.93 |
| Flatfish | 4.50 | 1.04 | 1.03 | 1.13 | 1.18 | 1.34 | 1.29 | 1.32 | 1.55 |
| Lanternfish and deep | 4.50 | 1.01 | 1.01 | 1.04 | 1.12 | 1.06 | 1.19 | 1.09 | 1.33 |
| Odontocetae | 4.54 | 0.00 | 1.01 | 0.00 | 0.99 | 0.00 | 1.01 | 0.00 | 1.03 |
| Orca | 5.50 | 1.01 | 1.00 | 1.01 | 0.98 | 1.01 | 0.97 | 1.01 | 0.96 |
|  |  |  |  |  |  |  |  |  |  |
| **Average by trophic level** |  |  |  |  |  |  |  |  |  |
|  | 1 | 0.99 | 0.00 | 1.14 | 0.00 | 1.12 | 0.00 | 1.10 | 0.00 |
|  | 2 | 1.14 | 1.27 | 0.94 | 1.17 | 1.05 | 1.32 | 1.04 | 1.57 |
|  | 3 | 0.94 | 1.02 | 0.92 | 1.02 | 0.93 | 1.10 | 0.93 | 1.19 |
|  | 4 | 0.90 | 1.34 | 0.91 | 2.75 | 0.94 | 3.89 | 0.95 | 5.96 |
|  | 5 | 1.01 | 1.00 | 1.01 | 0.98 | 1.01 | 0.97 | 1.01 | 0.96 |
