# Supplementary material for: Indirect Effects of Conservation Policies on the Coupled Human-Natural Ecosystem of the Upper Gulf of California
Source: PLoS One. 2013 May 15;8(5):e64085. doi: 10.1371/journal.pone.0064085 (PMC3654961; doi:10.1371/journal.pone.0064085)
Supplement: Text S1 — Northern Gulf of California model tuning and diagnostics. Excerpted from Ainsworth et al. [3]. (DOCX) [file pone.0064085.s010.docx]

In order to calibrate dynamic parameters in the Atlantis ecosystem simulation model for the Northern Gulf of California, it was necessary to compare the predicted dynamics of all ecosystem components with available historical time series of catch and relative abundance. We tuned dynamics of a historic model to available data, and then transferred the dynamic parameters to the present-day model assuming stationarity in feeding behavior and life history parameters of organisms [1].

We chose as a starting point for the historical model the year 1985 because we had the most comprehensive record of time series catch and relative abundance data from that point. In addition, it is the earliest available year provided by the regional ocean modeling system (ROMS) model. The development of the historic 1985 model for the Northern Gulf, the development of time series data from literature references, monitoring, and local environmental knowledge [2] is detailed in Ainsworth et al. [3].

The calibration process is iterative since the slow-run time in Atlantis prevents automated estimation of model parameters. Instead, state and rate parameters (i.e. recruitment variables, prey availabilities, predator consumption, mortality, and growth) are adjusted in order to generate realistic system behavior and fit predictions to observations. This overall strategy has been used in all Atlantis models built to date [4–7]. State and rate parameters were adjusted until extinctions no longer occurred under baseline fishing conditions and vertebrate numbers, body weights, and biomasses reached a stable equilibrium with reasonable values.

We ran a simulation without fishing, beginning from the 1985 model. Here we expected a slow increase in the biomass and abundance of exploited functional groups to some stable equilibrium level (unexploited biomass, B0, or numbers, N0). As expected, heavily exploited functional groups increase in biomass to a stable equilibrium (B0) upon release from fishing pressure. Responding to the increase in predator biomass, the prey base of the ecosystem is eroded while groups not heavily exploited maintain an equilibrium biomass close to their initial values. Individual weight at age (structural and reserve nitrogen) remained within an acceptable range for most functional groups and age-classes. A stable equilibrium forms for most groups with body weights ranging from 0.5 and 1.5 times initial values

The 1985 model was projected forward for 23 years to resemble the 2008 ecosystem condition using revised catch estimates (accounting for unreported catch and discards) and using the historical oceanographic forcing from a Regional Oceanographic Model System for 1985-2008. The Northern Gulf model recreated historical trends in species abundance, and observed ecological responses, as reported by monitoring, stock assessments, and local ecological knowledge (Figures A and B) [3]. Heavily exploited functional groups (e.g., Gulf coney, extranjero, leopard grouper, Gulf grouper, amarillo snapper, barred pargo, groupers and snappers, large reef fish, mojarra, mackerel) increase in biomass to a stable equilibrium (B0) upon release from fishing pressure. The result is less obvious in some highly aggregated functional groups in which only a fraction of species are heavily exploited (e.g., chano are contained in the aggregate group drums and croakers). Responding to this increase in predator biomass, the prey base of the ecosystem is eroded (e.g., small demersal fish, small reef fish). Groups not heavily exploited (e.g., macroalgae, sessile invertebrates) maintain an equilibrium biomass close to their initial values. Seasonality is also evident in the biomass plots; for example, large phytoplankton and small phytoplankton fluctuate according to availability of light and nutrients defined by the ROMS oceanographic inputs, while zooplankton shows an appropriate first-order reaction.

Figure A and B. Annual values of catch and biomass in simulation 1985–2008 (historical reconstruction scenario) for 48 functional groups. Black line shows biomass under historical fishing, shaded area shows catch. Start and end points (large circles) show 1985 and 2008 model biomass values, small circles show available time series data from stock assessment or surveys (residuals are minimized vs. predicted biomass). Historical oceanographic influences from ROMS are in effect. Figure is reproduced from Ainsworth et al. [3], originally Figure D-14 and D-15.

**
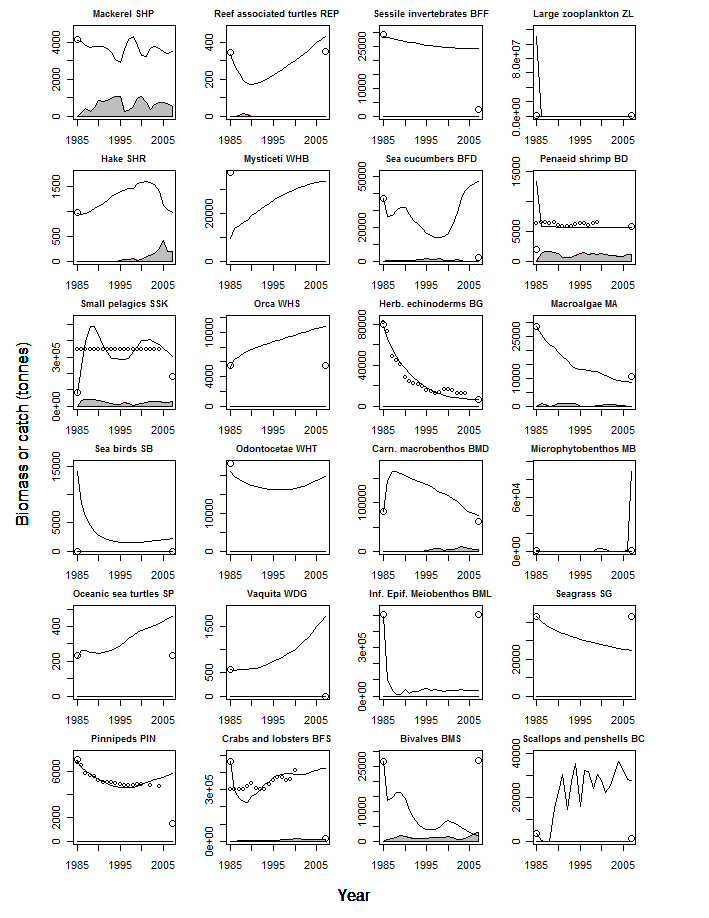

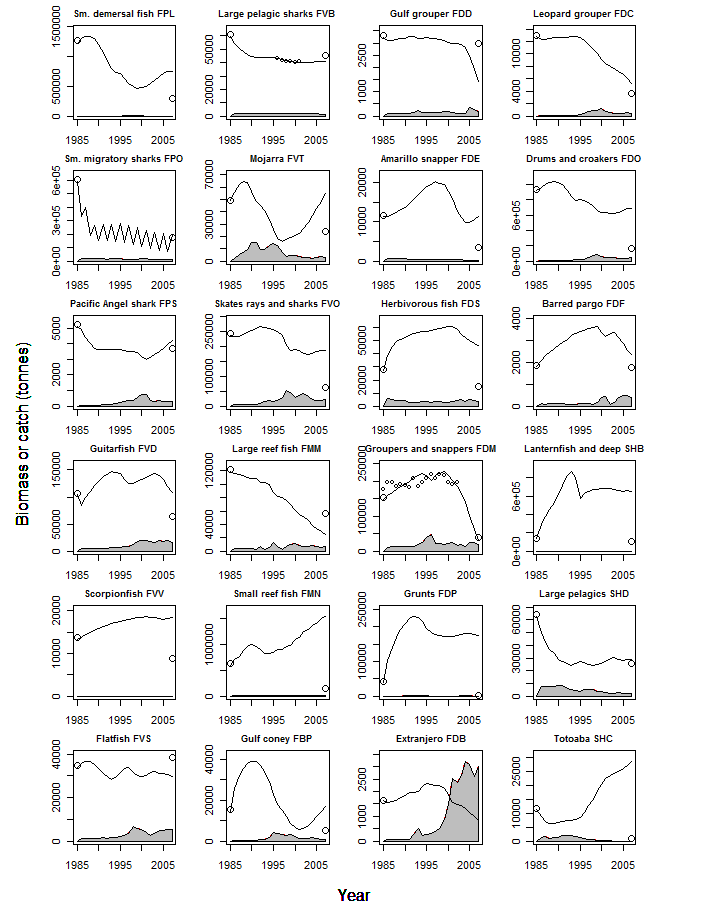
**

**References**

1. Ainsworth C, Kaplan IC, Levin PS, Cudney-Bueno R, Fulton EA, et al. (2011) Atlantis model development for the Northern Gulf of California. Seattle, WA: U.S. Department of Commerce. National Oceanic and Atmospheric Administration. National Marine Fisheries Service. NOAA Technical Memorandum NMFS-NWFSC-110. 293 p.

2. Ainsworth CH, Varkey D, Pitcher TJ (2008) Ecosystem simulations supporting ecosystem-based fisheries management in the Coral Triangle, Indonesia. Ecol Model 214: 361–374.

3. Ainsworth CH (2011) Quantifying species abundance trends in the Northern Gulf of California using Local Ecological Knowledge. Mar Coast Fish Dyn Manag Ecosyst Sci 3: 190 — 218.

4. Fulton EA, Parslow JS, Smith ADM, Johnson CR (2004) Biogeochemical marine ecosystem models. 2. The effect of physiological data on model performance. Ecol Model 173: 371–406.

5. Brand EJ, Kaplan IC, Harvey CJ, Levin PS, Fulton EA, et al. (2007) A spatially explicit ecosystem model of the California Current’s food web and oceanography. Seattle, WA: U.S. Department of Commerce. National Oceanic and Atmospheric Administration. NOAA Technical Memorandum NOAA TMNMFSNWFSC84. 163 p.

6. Horne PJ, Kaplan IC, Marshall KN, Levin PS, Harvey CJ, et al. (2010) Design and parameterization of a spatially explicit ecosystem model of the central California Current. Seattle, WA: U.S. Department of Commerce. National Oceanic and Atmospheric Administration. NOAA Technical Memorandum NMFS-NWFSC-104. 140 p.

7. Link JS, Fulton EA, Gamble RJ (2010) The northeast US application of ATLANTIS: A full system model exploring marine ecosystem dynamics in a living marine resource management context. Prog Ocean 87: 214–234. doi:10.1016/j.pocean.2010.09.020.
