# Supplementary material for: Indirect Effects of Conservation Policies on the Coupled Human-Natural Ecosystem of the Upper Gulf of California
Source: PLoS One. 2013 May 15;8(5):e64085. doi: 10.1371/journal.pone.0064085 (PMC3654961; doi:10.1371/journal.pone.0064085)
Supplement: Text S2 — Model-wide responses of biomass for species of conservation concern. (DOCX) [file pone.0064085.s011.docx]

Biomass responses (Figure 2 in main text) in the area affected by management actions (blue polygons in Figure 1, main text) were consistent with biomass responses calculated at the scale of the entire model, once spatial distribution of the species and fishing fleets [3] is taken into account. For instance, vaquita and sea lions show strong increases in abundance in the scenarios that add the most restrictions on fisheries (Primary Area Scenario and the Distribution Area Scenario). These increases are of similar magnitude whether calculated in the managed area or model-wide, because fleets with high sea lion and vaquita bycatch are concentrated in the managed area. Calculated within the managed area, species such as oceanic sea turtles and odontocetes show 5-10% increases in these two scenarios; model-wide these two groups show increases that are slightly lower because they are both found and caught within and outside the managed region. Species such as mysticetes, totoaba, and reef associate turtles generally showed smaller responses (< 4%, 3%, and 8% for the three groups respectively) to the management scenarios, and some of these responses vary when calculated for managed areas versus model-wide (Figure A). These species have large fractions of their populations outside the managed areas (mysticetes and reef associate turtles) and are still targeted outside the study area.

Figure A. Biomass for species of conservation concern under various management scenarios. Bars show percent change relative to the No vaquita management scenario in 2038. Results are for whole model extent.


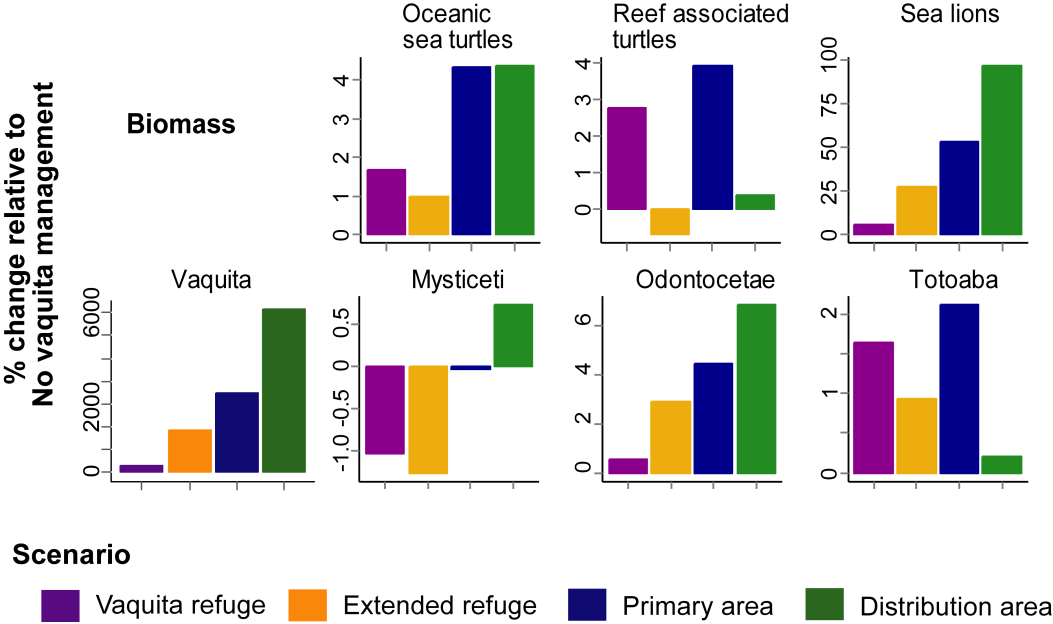


**References**

1. Ainsworth C, Kaplan IC, Levin PS, Cudney-Bueno R, Fulton EA, et al. (2011) Atlantis model development for the Northern Gulf of California. Seattle, WA: U.S. Department of Commerce. National Oceanic and Atmospheric Administration. National Marine Fisheries Service. NOAA Technical Memorandum NMFS-NWFSC-110. 293 p.
